# Grain Refinement of Inconel 718 Superalloy—The Effect of Rotating Magnetic Field

**DOI:** 10.3390/ma15062038

**Published:** 2022-03-10

**Authors:** Rui Pedro Silva, Rui Soares, Rui Neto, Ana Reis, Ricardo Paiva, Rui Madureira, José Silva

**Affiliations:** 1 INEGI—Institute of Science and Innovation in Mechanical and Industrial Engineering, Rua Dr. Roberto Frias, 4200-465 Porto, Portugal; rsoares@inegi.up.pt (R.S.); rneto@inegi.up.pt (R.N.); areis@inegi.up.pt (A.R.); rmp@inegi.up.pt (R.P.); rdmadureira@inegi.up.pt (R.M.); jmsilva@inegi.up.pt (J.S.); 2 Department of Mechanical Engineering, Faculty of Engineering, University of Porto, Rua Dr. Roberto Frias, 4200-465 Porto, Portugal

**Keywords:** investment casting, rotating magnetic field, grain refinement, Inconel 718, superalloy

## Abstract

The effect of the application of a rotating magnetic field on the average grain size of IN718 castings was experimentally studied. For the purpose, four parts were produced by investment casting and characterized. The first casting was produced without application of RMF for comparison. The remaining ones were submitted to different RMF frequencies for 15 min and subsequently to the pouring of the nickel-based superalloy. In these three castings, the RMF frequencies applied were, respectively, 15 Hz, 75 Hz and 150 Hz. All the other process parameters were kept constant during the execution of the experimental procedure. The average grain size of the samples was determined according to the ASTM E112-13 standard, using intercept methods. Macro hardness measurements, tensile testing and SEM-EDS analysis were conducted in order to evaluate the casting’s mechanical properties and microstructures. The results demonstrate a noticeable grain size reduction in the samples submitted to rotating magnetic field. An average grain area reduction, greater than 96%, was achieved in the castings where RMF frequencies of 75 Hz and 150 Hz were applied. The application of RMF also caused a morphological change in the casting’s dendrites from cellular to almost equiaxed. Additionally, it originated the decrease of the size and amount of needle-like δ phase. Regarding mechanical properties of the cast parts, no major differences were verified.

## 1. Introduction

Inconel (Nickel-based) superalloys are currently used in the aircraft/aerospace industry, mostly due to their excellent creep properties, hot corrosion resistance and oxidation resistance. These superalloys can withstand intense mechanical stresses and pressures, when submitted at high working temperatures (heat resistant alloys), while retaining reliable creep and corrosion-resistance properties [1,2,3,4].

Therefore, Inconel 718, a Ni-Cr-Fe austenite (γ) superalloy [1,2,5] has been mostly employed in turbojet engines in the form of major parts of turbines wheel, discs and blades. It is also used in rocket engines, combustion chambers and nuclear reactors as it offers robustness and prevents the occurrence of brittle transformation in the components at high temperatures [1,2,3,4,5,6,7].

At high operating temperatures, normally up to 700 °C, it is desirable as a uniform and fine crystal structure of the superalloy in order to preserve its excellent mechanical characteristics [5,8].

Nickel-based superalloys can also be hardened by solid-solution strengthening or precipitation of intermetallic compounds in the metal matrix. IN718, in particular, is frequently strengthened by precipitating γ’ (Ni_3_(Al, Ti)) and γ’’ (Ni_3_Nb) phases in the γ matrix [9,10,11,12,13,14].

Another important precipitated phase noticeable in Inconel 718 is δ phase (orthorhombic Ni_3_Nb), which is incoherent with the γ matrix. The precipitation of acicular δ phase often starts at 750 °C and occurs up to 1020 °C, influencing the mechanical properties of the superalloy. Despite the fact that the significant presence of δ phase at grain boundaries might drastically decrease the strength and plasticity of the alloy, the moderate presence of δ phase could promote grain refinement and inhibit its dislocation (δ phase grain boundary pinning effect—spherical fine δ phases), improving the mechanical properties of Inconel 718 [2,9,10,15]. Typically, the needle-like δ phase forms in the vicinity of or is attached to the Laves phase, mainly in the Nb-rich areas of the interdendritic regions [13]. Laves phases have low ductility and their greater incidence causes a decrease in castings’ mechanical and corrosion properties [13,14,15,16].

Usually, Inconel 718 components are fabricated using conventional melting and investment casting techniques. Nevertheless, other processes that allow one to obtain components with complex shape and highly precise dimensions, such as selective laser melting (SLM), are also applied to produce these parts [10,11,14].

The major drawbacks of conventional investment casting of superalloys such as Inconel are microstructural coarseness and non-uniformity of grain size, which could negatively affect the performance and reliability of cast turbine components operating at medium/high temperatures [2,17]. Other melt-related problems that lead to a degradation of the mechanical properties of Inconel superalloys are segregation, porosities and laves phase [9,13,18]. Therefore, additional casting techniques are needed to produce very fine grains and homogenous microstructures.

Nowadays, three major casting techniques are applied aiming grain refinement of superalloys, such as [8,19]:

- Variation of casting parameters (namely by controlling the casting temperature and the heat transfer at the interface metal/mould);
- Addition of grain refinement agents (Inoculants, e.g., CoAl_2_O_4_);
- Mechanical vibration.

These methods can be grouped into three distinct categories: thermal, chemical and mechanical.

For example, J. Wenzhong et al. [8] state that fine-grain superalloy castings with a grain size in the range of 125–65 µm might be achieved by controlling the cooling rate (rapid cooling preferable), adding inoculants and simultaneously agitating the mould. This agitation of the mould might be performed by submitting the liquid metal to electromagnetic stirring by the imposition of a rotating magnetic field [8].

Electromagnetic stirring is an effective method to obtain grain refinement and homogenous refining structure [9]. Its principle is based on the application of a system of two fields: an electric and a magnetic one. These fields exhibit a mutual relationship described by the *Maxwell* equations [20].

The induction coil, powered by electrical current (I_0_), generates an electromagnetic field that interacts with the solidifying metal at the mould. Hence, this electromagnetic field induces a local electromotive force (*E_m_*) with a magnitude dependent on the local speed of the liquid metal (*V*) and magnetic induction (*B*) [20]:

(1) $$E_{m}=\bar{V}\times \bar{B}$$

This vector product is a result of the interaction between the magnetic field lines and the current flow of the liquid metal. Consequently, an *eddy* current (*I*) is induced in the liquid metal (conductor) [20]:

(2) $$\bar{I}=\sigma \left(\bar{V}\times \bar{B}\right),$$

where *σ* represents the electrical conductivity of the liquid metal.

The *Lorentz* force (*F*) is a consequence of the effect of the induced current on the magnetic field [20]:

(3) $$\bar{F}=\bar{I}\times \bar{B}$$

The *Lorentz* force is responsible for the generation of a torque that gives the liquid metal a rotational movement with the same direction of the rotating magnetic field. The generated torque depends mainly on the intensity of supply current, frequency, number of coil’s windings and system geometry. When the vectors V and B are perpendicular, the *Lorentz* force has is maximum magnitude [20].

Finally, the induced current intensity and the speed of the liquid metal in motion might be related using *Ohm*’s law [20]:

(4) $$\bar{I}=\sigma \left[\bar{E}+\left(\bar{V}\times \bar{B}\right)\right]$$

**Figure 1:**
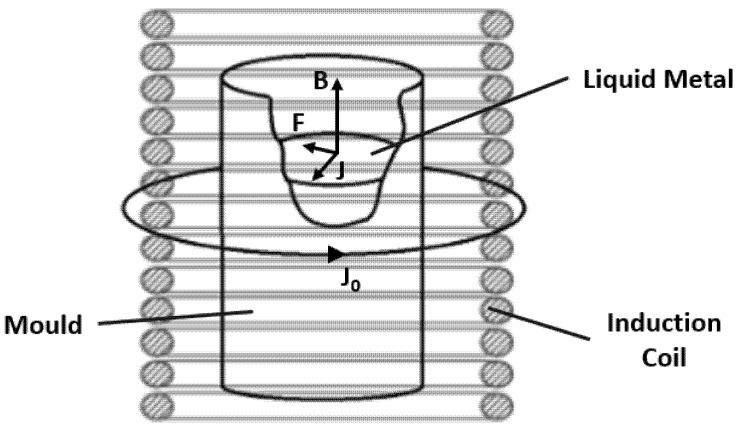
Influence of the electromagnetic field on the liquid metal (J represents the current density).

Figure 1 displays the influence of the magnetic field on the liquid metal within the mould and emphasizes the relationship between the physical quantities expressed in the last paragraphs.

Electromagnetic stirring increases the fraction of equiaxed grains [21]. For example, the forced liquid metal movement promotes the transportation of the crystals from the mould wall into the liquid metal, where they can be converted in equiaxed crystals [8,20]. Additionally, it reduces the segregation and the formation of shrinkage cavity. This method is essential for grain refinement of superalloys since ease nucleation is promoted while an extensive growth of crystals in the melt is avoided (it decreases the velocity of columnar crystals growth) [17,20,22].

In summary, the strong convection of liquid metal caused by the rotating magnetic field modifies its velocity distribution in the melt and changes its solidification behaviour. The magneto–fluid dynamics affect the solid–liquid growing mode and inhibits the segregation of alloying elements on the front of solid–liquid interface. These effects reduce metallurgical defects, resulting in finer microstructures and enhanced mechanical properties [9,21,22].

Moreover, coating the internal surface of the ceramic shells with inoculants results in the refinement of the surface grains of superalloy castings (increasing of heterogeneous nucleation sites and grain growth hindering) [8,17,23]. Along with pouring temperature, the presence of inoculants in the facecoat of the mould plays a significant role in grain size control. One of the most used inoculants in the investment casting process of Nickel-based superalloys is cobalt aluminate (CoAl_2_O_4_) [19,23].

As mentioned previously, Inconel 718 is highly sensitive to metallurgical defects such as porosities, coarse grain sizes and segregation, which might decrease the mechanical performance of the structural components produced with this superalloy [18,24].

Therefore, when used in structural components, investment cast IN718 is hot isostatically pressed (HIP treatment) in order to reduce porosities due to alloy contractions and casting segregation [6,13,24]. In addition to mechanical vibration, HIP treatment is an important technique to decrease IN718 casting defects by promoting a homogenous and dense microstructure and enhancing the mechanical properties of the alloy [6,13,18]. By simultaneously applying high temperature and high gas pressure to the parts, the HIP process enables the uniform density of the castings, with a significant decrease of porosity and resulting in improved creep, fatigue, ductility and tensile properties [13,18,24].

In the present study, the application of a rotating magnetic field on the average grain size, microstructure and mechanical properties of IN718 castings will be investigated.

## 2. Materials and Experimental Procedure

### 2.1. Materials

The superalloy used in the experimental work was Inconel 718. Its chemical composition (weight %) is presented in Table 1. This alloy owns a melting point in the range 1210–1344 °C. A cylindrical ingot (ø 73 mm; height 164 mm) with 5.6 kg was used for the investment casting process.

### 2.2. Investment Casting Process

**Figure 2:**
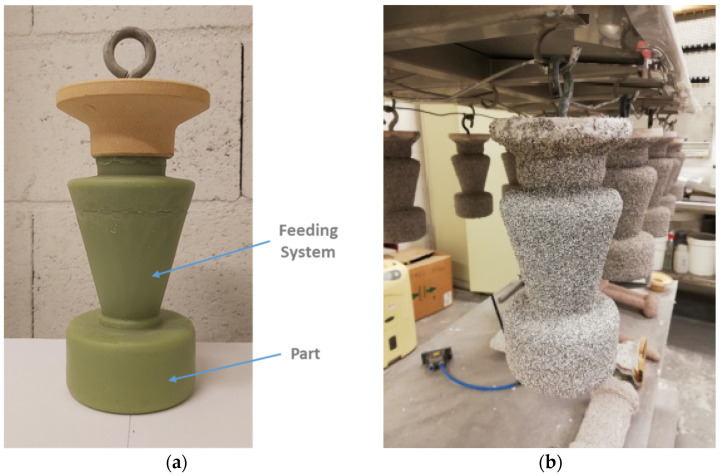
Initial stages of investment casting: (**a**) wax pattern produced; (**b**) ceramic shell (appearance after 5 of 9 coatings).

The part to be produced by investment casting consists of a cylinder with 95 mm diameter and 50.95 mm height. Furthermore, a conical feeding section was added to the top of the part to ensure an enhanced filling of the ceramic shell. The mentioned geometry can be seen in Figure 2a.

Subsequent to the production of the wax patterns and ceramic moulds, dewaxing and ceramic shell heating process, the casting process was conducted following the seven stages described below:

1. Pre-heating of the ceramic shells (T = 1000 °C), crucible and inconel’s ingot (T = 250 °C);
2. Placement of the shell, crucible and metallic ingot in the casting equipment;
3. Vacuum application inside the casting chamber (pressure: 0.1 mbar);
4. Inconel’s ingot melting initiation;
5. Pouring of the metal into the ceramic shell (by gravity effect);
6. EMS application (duration: 15 min);
7. Air cooling of the ceramic shell.

**Figure 3:**
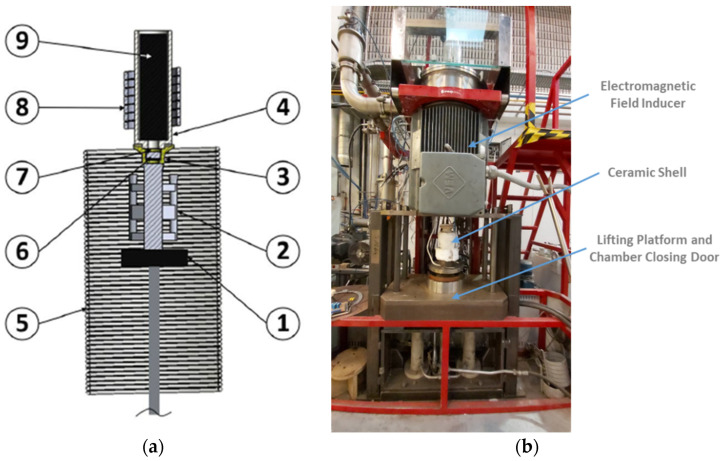
Equipment used for investment casting and application of electromagnetic field: (**a**) schematic representation of the equipment and identification of the main components; (**b**) picture of the equipment used in the experiments.

In order to prevent a shell’s faster cooling and to provide more time to metal’s grain size refinement through EMS, a thermal blanket (superwool) was applied around the ceramic shells previously to the casting process. Figure 3a schematizes the equipment used to perform the castings and to generate the electromagnetic field. This equipment is composed of a lifting platform (1), an electromagnetic field inducer (5) and an induction coil (8). In this figure, the position of the ceramic shell (2), pouring cup (3), ceramic crucible (4), ceramic filter (6), nickel penny (7) and the Inconel ingot (9), inside the chamber when the lifting platform is raised and the door closed, is visible.

During the process, after the raise of the lifting platform and subsequently close of the chamber door, vacuum was applied in the casting chamber before the Inconel ingot melting (vacuum melting start value: 0.1 mbar), aiming to avoid the occurrence of oxidation reactions.

In order to study the effect of the rotating magnetic field in the grain refinement of Inconel 718 and its microstructure, four parts were produced with different electromagnetic field frequencies, according to Table 2. For this purpose, a frequency inverter *MOVITRAC LTP-B* was used in the connection between the equipment’s stator and the electrical network. A rotating magnetic field without reversion was applied. The remaining process parameters were kept constant during the execution of the experimental procedure. The current intensity applied represents the maximum value that allows one to use the equipment without stator’s overheating.

### 2.3. Casting’s Characterization

The cylindrical cast parts obtained (without the upper conical feeding system) were cut into six slices by electrical discharge machining (EDM). In order to evaluate the influence of the rotating magnetic field on the cast parts, average grain size measurements were performed, followed by a microstructural and mechanical properties analysis.

The determination of the average grain size was executed according to the ASTM E112-13 standard (intercept method—40 mm test line length). For each casting, this method was applied both in a section from the center of the cast part and in another from its periphery. Three measurements were performed for each section. The samples were ground, polished and chemically etched with 80 mL HCL + 10 mL H_2_O_2_ + 10 mL H_2_O to expose the macrostructure.

Microstructures were characterized using a FEI Quanta 400FEG ESEM high resolution scanning electron microscopy (SEM) (FEI Company, Hillsboro, OR, United States) equipped with an EDAX Genesis X4M energy-dispersive x-ray spectroscopy microanalysis (EDS)(Oxford Instruments, Oxfordshire, UK). In order to expose the different intermetallic compounds, the samples were ground, polished and chemically etched with 60 mL C_2_H_5_OH + 40 mL HCL + 2 g CuCl_2_.

**Figure 4:**
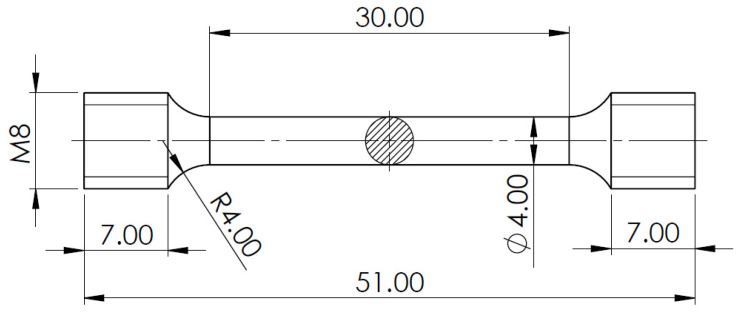
Sketch of standard tensile testing specimen (dimensions in mm).

Mechanical properties were analyzed by macro hardness measurements and tensile testing. The macro hardness measurements were performed using a EMCO M4U Universal Hardness Tester (EMCO-Test, Kuchl, Austria). Three measurements were done for each casting, in the section closest to the center of the cast part. The tensile tests were accomplished using an MTS 810 Material Testing System. For each casting, three specimens were machined from the section closest to the center of the cast part to prepare standard tensile samples (ISO 6892-2). The specimens’ dimensions can be visualized in Figure 4.

## 3. Experimental Results and Discussion

### 3.1. Grain Size Evaluation

The grain measurement results obtained for the castings submitted to four distinct RMF frequencies are presented in Table 3. The number of intercepts per unit length of test line ($\bar{N_{L}}$), the mean lineal intercept length ($\bar{l}$) and the ASTM macroscopic grain size number (*G*) were calculated, respectively, using the following equations, in accordance with ASTM E112-13 standard:

(5) $$\bar{N_{L}}=\frac{N_{I}}{L/M}$$

(6) $$\bar{l}=\frac{1}{\bar{N_{L}}}$$

(7) $$G=+10.00-2\times \log_{2}\bar{l}$$

where *L* represents the test line length (40 mm) and M is the magnification used (1× in this case).

The number of grains per unit area ($\bar{N_{A}}$ [No./mm^2^]) and the average area of the grain sections ($\bar{A}$ [mm^2^]), for macroscopically determined grain sizes, can be obtained using the following relation with ASTM grain size (G):

(8) $$G=-2.9542+3.3219\times \log_{10}(\bar{N_{A}}\times 100^{2})$$

So,

(9) $$\bar{N_{A}}=10^{\frac{G+2.9542}{3.3219}}/100^{2}$$

And,

(10) $$\bar{A}=1/\left(\bar{N_{A}}\right)$$

The results regarding the number of grains per area unit and the average area of the grain sections, for each casting, are exhibited in Table 4. As can be perceived from these results, the grain size was highly reduced from the first casting (without RMF application) to the other ones submitted to rotating magnetic field. Additionally, for all the four castings, the average grain size measured was smaller in the center of the cast part rather than in its periphery. That was unexpected for casting no. 1, produced without RMF, since the early solidification of the periphery means faster cooling time and, consequently, should have resulted in finer grain size in this section. Nevertheless, due to the small number of grains intercepted by a test line in the samples of the first casting, the difference in the grain size measured in the central and peripheral sections can be neglected. In the samples submitted to RMF, this fact can be explained due to the earlier cooling and subsequent solidification of the castings’ peripheral sections when compared to the central ones since the periphery has less time to engage in inconel’s grain size refinement through electromagnetic stirring.

**Figure 5:**
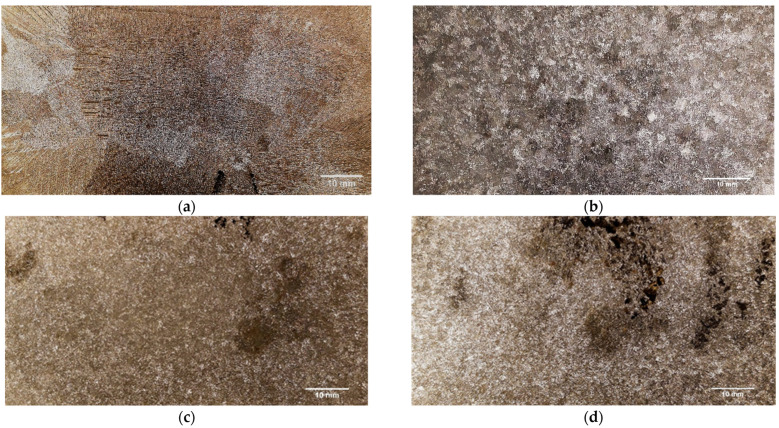
Grain Size in the center of the cast parts: (**a**) coarse grain—casting no. 1 (without RMF application); fine grain—(**b**) casting no. 2 (15 Hz); (**c**) casting no. 3 (75 Hz); and (**d**) casting no. 4 (150 Hz).

For the section closest to the center of the cast part, the major difference noticed was between casting no. 1 and 4, with a reduction of 96.70% in the average grain area. A similar grain size reduction was obtained for casting no. 3 (96.65%). Despite this, casting no. 4 in its central section presented a great number of casting defects due to shrinkage porosity. These casting defects can be observed in Figure 5d.

Regarding the section furthest from the center of the cast part, the main decrease in the average grain area was among casting no. 1 and 3 (96.82% reduction). Amongst the castings submitted to RMF, the highest decrease in the average grain area was achieved between casting no. 2 and 3 (49.92% reduction for the peripheral section of the cast part).

**Figure 6:**
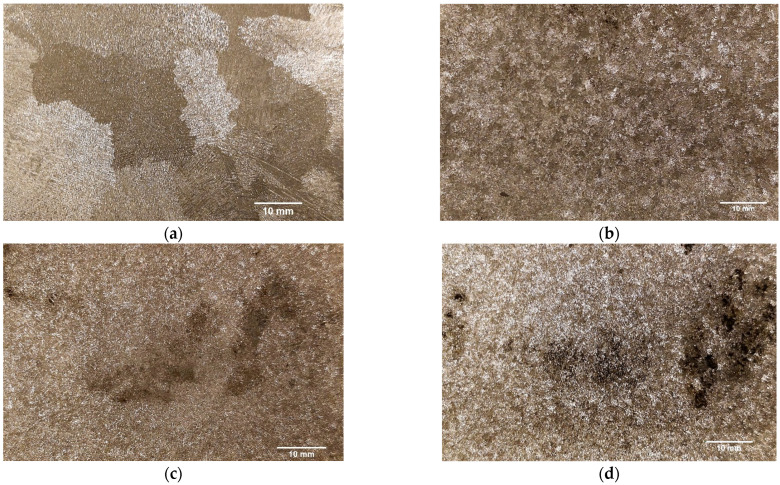
Grain size in the periphery of the cast parts: (**a**) coarse grain—casting no. 1 (without RMF application); fine grain—(**b**) casting no. 2 (15 Hz); (**c**) casting no. 3 (75 Hz); and (**d**) casting no. 4 (150 Hz).

The greatest overall results were obtained for casting number 3 (rotating magnetic field frequency: 75 Hz). The grain size refinement that occured due to the application of rotating magnetic field can be noticed at Figure 5 and Figure 6, where the macrostructures of the four different castings are presented, in their central and peripheral sections, respectively. From the visualization of these pictures, it is possible to verify the enormous difference between the grain size of castings no. 1 (coarse grain) and the grain size of the castings submitted to RMF (fine grain).

**Figure 7:**
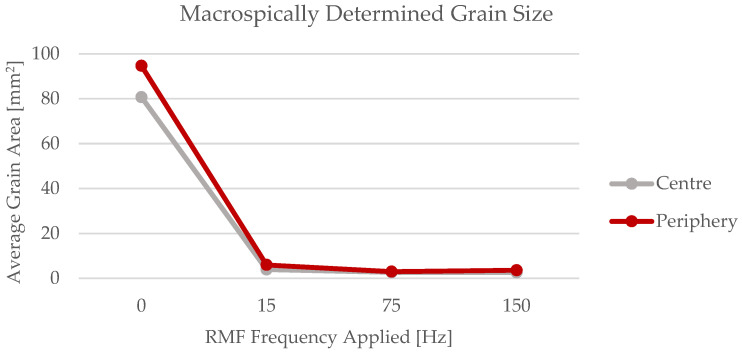
Evolution of the average grain size with the RMF frequencies applied.

The evolution of the average grain area with the rotating magnetic field frequencies applied can be visualized in Figure 7. In this figure, the major dissimilarity in the grain size between the casting produced without magnetic field application and the ones submitted to rotating magnetic field is noticeable. The results achieved prove that the forced liquid metal movement caused by the RMF, during the beginning of the casting’s solidification, effectively contributed to grain refinement.

### 3.2. Microstructures and Metallographic Evaluation

**Figure 8:**
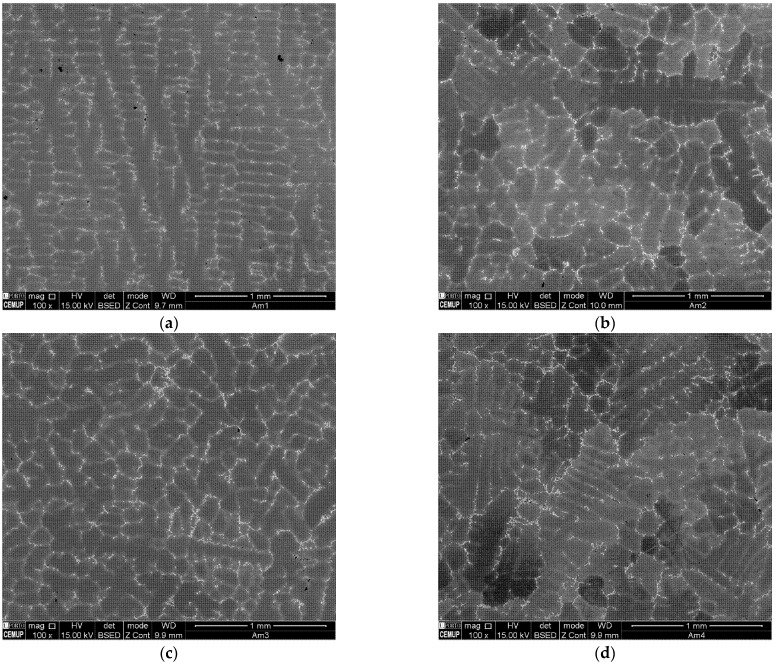
Backscattered SEM images of each cast part produced: (**a**) casting no. 1 (0 Hz); (**b**) casting no. 2 (15 Hz); (**c**) casting no. 3 (75 Hz); and (**d**) casting no. 4 (150 Hz).

Figure 8 displays the backscattered SEM images of the four castings produced. From its analysis, the morphological change caused by the application of the rotating magnetic field is noticeable. Therefore, at casting no. 1 sample, produced without application of RMF, dendrites with cellular morphology are predominant (Figure 8a). On the other hand, in the samples representative of the three castings submitted to RMF, the dendrites have an equiaxed morphology.

As characteristic of cast IN718 [14], extensive segregation is observed in the microstructures of the castings produced, mainly in the interdendritic space but also in the dendritic (γ) region. This segregation is intensified due to the large section size of the cast parts since longer solidification process occurs [13].

**Figure 9:**
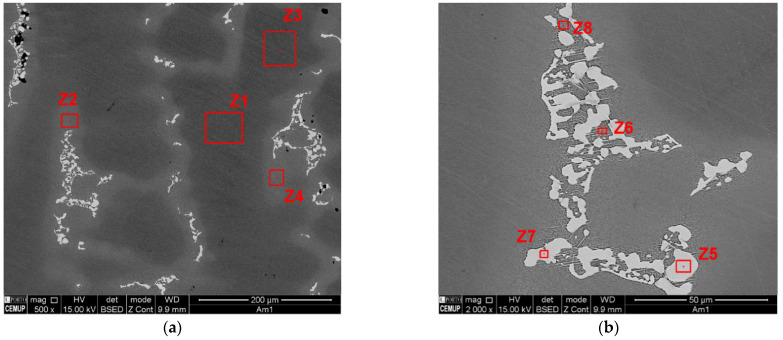
Phase distribution and identification in as-cast IN718 (Casting no. 1): (**a**) Overview of the dendritic (Z1 and Z3) and interdendritic (Z2 and Z4) regions; (**b**) Phases identification within interdendritic region.

Figure 9 exhibits the phase distribution in the as-cast IN718 without application of RMF. Subsequent to the nucleation of the γ phase, the initial phase formed is the Nb and Ti primary carbides [13]. Despite the fact this primary carbide is scattered throughout the matrix (Z1 and Z3), it is more predominant in the Nb rich interdendritic regions (Z2 and Z4). The interdendritic phases are principally Laves (Z5 and Z7) and Carbides—MC (Figure 11-Z9). Nevertheless, as the cast parts’ temperature decreased, a needle-like phase called delta (orthorhombic Ni_3_Nb) formed in the close vicinity of the Laves phase (Z6 and Z8). Additionally, cooling resulted in the formation of γ’’ precipitates, smaller plate-like structures that represent a metastable form of Ni_3_Nb [9,18]. In the interdendritic regions, porosities are also noticeable, mostly associated with Laves islands. 

**Figure 10:**
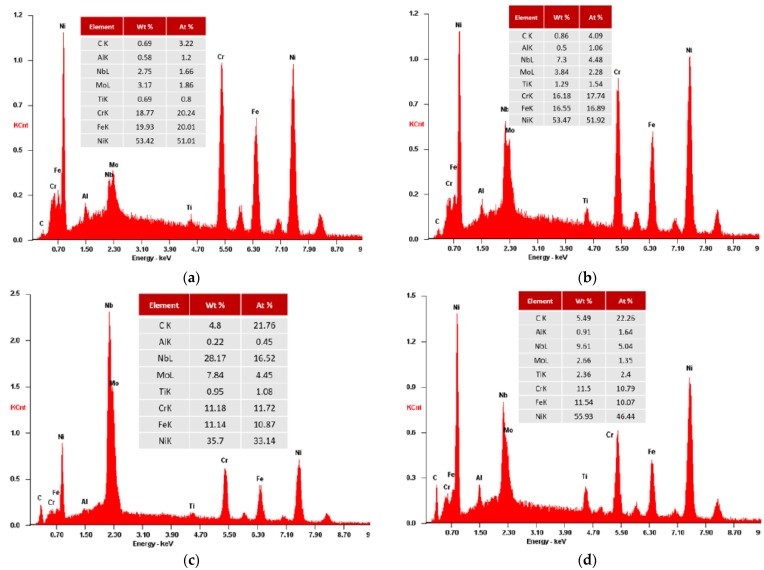
EDS analysis results for as-cast IN718 (Casting no. 1): (**a**) Dendritic region (Z1 and Z3); (**b**) Interdendritic region (Z2 and Z4); (**c**) Laves phase (Z5 and Z7); and (**d**) Delta phase plus γ’’ (Z6 and Z8).

The SEM-EDS analysis of the as-cast Inconel 718 (Figure 10) confirms a low Nb content, about 2–3% (wt%), in the dendritic matrix, and a higher Nb content, greater than 7% (wt%), in the interdendritic region. It is also perceptible that the segregation areas of the interdendritic region are enriched with Nb, Ti and Mo but have reduced concentrations of Fe and Cr when compared to the dendritic core. The Nb content of the Laves phase is, approximately, 28% (wt%). Regarding δ phase, its Nb content is 8–10% (wt%).

Niobium plays a fundamental role in achieving chemical and mechanical property uniformity in IN718 castings. IN718 cast alloys with Nb contents greater than 5% might require extended homogenization treatments, in order to obtain optimized mechanical properties, since the uniform precipitation of fine γ’ and γ’’ phases requires dissolution of the Laves phase along with Nb interdiffusion.

Based on the state-of-art analysed, the amount of segregation and the presence of shrinkage porosities can be decreased with the execution of HIP treatments [18,24].

**Figure 11:**
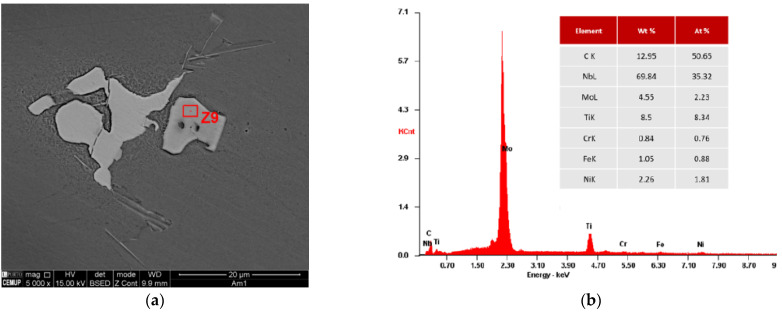
Identification of a MC carbide present in as-cast IN718 (Casting no. 1): (**a**) SEM image; (**b**) EDS analysis and chemical composition determination.

Figure 11 displays the SEM visualization of a MC carbide and its respective composition obtained by EDS analysis. Due to the high Nb concentration, almost 70% (wt%), it is most likely to be a NbC carbide. 

**Figure 12:**
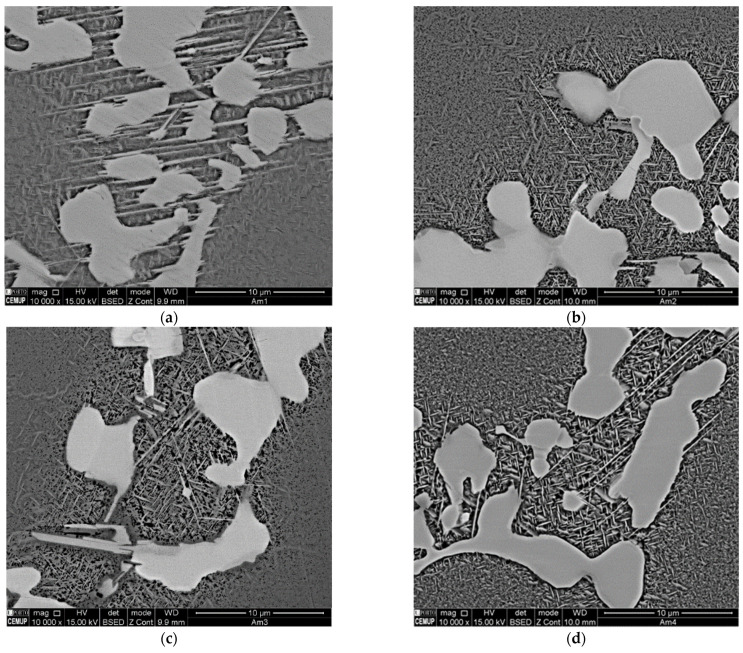
Influence of RMF on delta phase size and amount: (**a**) Casting no. 1 (without RMF); (**b**) Casting no. 2 (RMF Frequency 15 Hz); (**c**) Casting no. 3 (RMF Frequency 75 Hz); and (**d**) Casting no. 4 (RMF Frequency 150 Hz).

Similar phase composition and distribution within the dendritic or interdendritic areas were found in the four castings produced. The major difference is exposed when comparing the SEM images of casting no. 1, produced without application of rotating magnetic field, with the ones of the castings submitted to RMF, i.e., the needle-like δ phase (orthorhombic Ni_3_Nb) suffered an evident reduction of size and quantity with the application of RMF (Figure 12). In the samples where rotating magnetic field was applied, the precipitation of the γ’’ smaller plate-like phases seemed to be pronounced.

### 3.3. Mechanical Properties

The mechanical properties achieved for the samples regarding the four different castings produced were rather similar.

Due to the great amount of casting defects (shrinkage porosities) present in the central section of casting number 4 (RMF frequency 150 Hz), it was not possible to produce enough valid specimens for mechanical properties characterization of this casting. According to the literature review performed, these casting defects could be minimized with application of a HIP treatment, where the cast IN718 is hot isostatically pressed in order to reduce the porosities generated due to alloy contractions [18,24].

Therefore, the mechanical properties achieved for the casting produced without RMF application, and subsequently with coarser grain size, were only compared with the ones attained for the casting submitted to rotating magnetic field where the enhanced grain refinement was accomplished.

**Figure 13:**
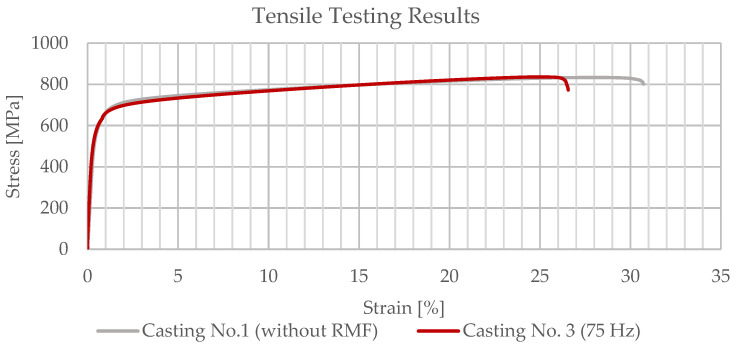
Tensile testing results for casting no. 1 (specimen 3) and casting no. 3 (specimen 1).

The engineering stress-strain curves obtained for a specimen of casting number 1, not submitted to rotating magnetic field, and a specimen of casting number 3, submitted to a rotating magnetic field with a frequency of 75 Hz, are presented at Figure 13. Despite the great dissimilarity in the average grain size results attained for these two castings (casting no. 1—coarser grain with average grain area of 80.64 mm^2^; casting no. 3—finest grain with average grain area of 2.70 mm^2^ in the central section of the casting), there seem to be no major differences in their mechanical properties, as might be expected from the tensile testing results.

For the casting produced without the application of the RMF, a yield strength (Rp_0.2_) of 575 MPa, an ultimate tensile strength (R_m_) of 828 MPa and an elongation (A_5,01_) of 29.8% were achieved. Similar to these results, for casting number 3 (RMF frequency 75 Hz), a yield strength (Rp_0.2_) of 575 MPa, an ultimate tensile strength (R_m_) of 833 MPa and an elongation (A_5,01_) of 27.5% were obtained. The results denoted in this paragraph are exhibited in Table 5.

From the tensile testing results achieved for both castings number 1 (without RMF) and 3 (RMF frequency 75 Hz), it might be concluded that the application of electromagnetic stirring during the beginning of the casting solidification did not significantly affect its mechanical properties. Since the use of RMF significantly contributed to the reduction of the average grain size of the castings, it was expected that the castings submitted to RMF possess improved mechanical properties. Nevertheless, the existence of shrinkage porosities in the center of the cast parts due to its great massiveness might have influenced the tensile testing results. The presence of these defects negatively affected the mechanical properties of the specimens extracted from the central section of the castings, preventing one from differentiating between the results from the samples with and without RMF application.

The macro hardness measurements results are in line with the tensile testing ones. For the four different castings produced, the average hardness accomplished was quite similar. These results are presented in Table 6.

**Figure 14:**
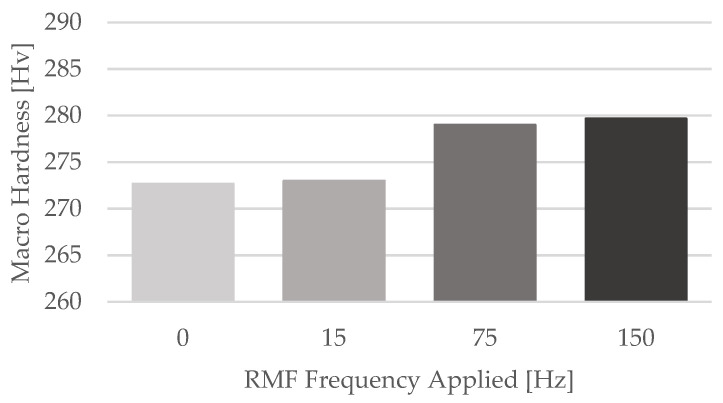
Average Macro Hardness results for each casting.

Comparing the average macro hardness results attained for each one of the four castings, a slight increase of its value with the increment of the RMF frequency is noticeable. Despite this evidence, the major increase on the average macro hardness was only about 2.57% (amongst casting no. 1 and casting no. 4), which can be neglected. The comparison between the average macro hardness results obtained for the four castings submitted to different RMF frequencies is displayed at Figure 14.

## 4. Conclusions

The effects of the application of a rotating magnetic field on the average grain size, microstructures and mechanical properties of Inconel 718 castings were investigated in this study. Therefore, four cast parts were produced by investment casting: the first one without application of rotating magnetic field and the remaining ones submitted to distinct RMF frequencies of 15 Hz, 75 Hz and 150 Hz, respectively. Based on the research developed, several conclusions can be summarized as follows:

1. The application of rotating magnetic field subsequent to the pouring of IN718 significantly contributed to the reduction of the average grain size of the castings. Therefore, the results accomplished demonstrate that the forced liquid metal movement during casting’s solidification caused by the RMF effectively generates grain refinement.
2. An average grain area decrease greater than 96% was achieved in the castings where RMF frequencies of 75 Hz and 150 Hz were applied. The greatest reduction (96.82%) was attained in the peripheral section of casting no. 3 (RMF frequency: 75 Hz), which represents a grain area decrease from 94.64 mm^2^ to 3.01 mm^2^.
3. The application of RMF caused a morphological change in the cast parts: at casting no. 1, produced without application of RMF, dendrites with cellular morphology are predominant; in the remaining three castings, submitted to RMF, dendrites’ morphology is mainly equiaxed.
4. Regarding the microstructural evaluation, similar phase composition and distribution within the dendritic and interdendritic areas were visualized in the four castings produced. The major dissimilarity perceived in the samples submitted to RMF was the evident decrease in size and quantity of the needle-like δ phase (orthorhombic Ni_3_Nb). Additionally, in these samples, the precipitation of the γ’’ smaller plate-like phases seems to be pronounced.
5. Concerning the mechanical properties of the cast parts, no major differences were observed in the tensile testing and macro hardness measurements performed in the specimens of the castings submitted, or not, to rotating magnetic field. Therefore, the application of RMF during the beginning of the castings’ solidification appears not to have significantly affected their mechanical properties.
6. Since the application of rotating magnetic field vastly contributed to the reduction of the average grain size of the cast parts and caused the reduction of the needle-like δ phase, which is responsible for the decrease of strength and ductility of IN718, it was expected that the mechanical properties of the castings would increase due to RMF. However, as pointed out in the previous paragraph, that was not verified in the tensile testing results. This effect might be related to the great size of the castings, which contributes for the generation of shrinkage porosities in its center, the section from where tensile testing specimens were extracted. The presence of these defects negatively affected the mechanical properties of the specimens, preventing one from differentiating between the results from the samples with and without application.
7. Due to the great size of the cast parts, most of the samples produced presented shrinkage porosities. Casting no. 4 (RMF frequency 150 Hz) was the one with the greatest number of these casting defects, mainly in its central section. According to the state-of-art, these casting defects could be minimized with the execution of a HIP treatment, where the IN718 cast parts would be hot isostatically pressed in order to reduce the porosities generated due to alloy contractions.

## Figures and Tables

**Table 1:** Chemical composition of Inconel 718 alloy (wt %).

**Element**	**C**	**Mn**	Si	Ti	Cu	Al	Co	Mo	Nb	Fe	Cr	Ni
**Standard** **composition (%)**	≤0.08	≤0.35	≤0.35	0.65–1.15	≤0.30	0.20–0.80	≤1.0	2.80–3.30	4.75–5.50	Bal	17.00–21.00	50.00–55.00
**Actual** **composition (%)**	0.032	0.015	0.073	0.871	0.0093	0.65	0.045	3.01	5.36	19.59	18.09	52.00

**Table 2:** DoE implemented to study the effect of rotating magnetic field application during the investment casting of Inconel 718.

Casting Number	Frequency (Hz)	RMF Current Intensity (A)	RMF Treatment Time (s)	Power (kW)
**1**	0 *	-	-	-
**2**	15	80	900	65
**3**	75	80	900	65
**4**	150	80	900	65

* Casting number 1—rotating magnetic field not applied.

**Table 3:** Grain measurement results for the different castings.

Casting Number	RMF Frequency (Hz)	Section	Test Line No.	Ni	$\bar{N_{L}}$	$\bar{l}$(mm)	${\bar{l}}_{\mathrm{Section}}$ (mm)	ASTM Macro Grain Size-G
**1**	without magnetic field	Center	1	5	0.125	8.00	**8.00**	**M-4.00**
			2	5	0.125	8.00		
			3	5	0.125	8.00		
		Periphery	1	4	0.100	10.0	**8.667**	**M-3.77**
			2	5	0.125	8.00		
			3	5	0.125	8.00		
**2**	15	Center	1	23	0.575	1.74	**1.771**	**M-8.35**
			2	24	0.600	1.67		
			3	21	0.525	1.91		
		Periphery	1	19	0.475	2.11	**2.183**	**M-7.75**
			2	18	0.450	2.22		
			3	18	0.450	2.22		
**3**	75	Center	1	26	0.650	1.54	**1.465**	**M-8.90**
			2	28	0.700	1.43		
			3	28	0.700	1.43		
		Periphery	1	24	0.600	1.67	**1.545**	**M-8.75**
			2	26	0.650	1.54		
			3	28	0.700	1.43		
**4**	150	Center	1	28	0.700	1.43	**1.454**	**M-8.92**
			2	30	0.750	1.33		
			3	25	0.625	1.60		
		Periphery	1	25	0.625	1.60	**1.702**	**M-8.47**
			2	25	0.625	1.60		
			3	21	0.525	1.91		

Where: Ni—number of intercepts; $\bar{N_{L}}$—number of intercepts per unit length of test line; $\bar{l}$—mean lineal intercept length for each test line; and ${\bar{l}}_{\mathrm{Section}}$—average of mean lineal intercept length for each section.

**Table 4:** Grain size determination for the different castings.

Casting No.	RMF Frequency (Hz)	Section	$\bar{N_{A}}$ (No./mm^2^)	$\bar{A}$ (mm^2^)	Grain Size Reduction * (%)
1	without magnetic field	Center	0.0124	80.64	-
		Periphery	0.0106	94.64	-
2	15	Center	0.2533	3.95	95.10
		Periphery	0.1665	6.01	93.65
3	75	Center	0.3697	2.70	96.65
		Periphery	0.3327	3.01	96.82
4	150	Center	0.3754	2.66	96.70
		Periphery	0.2741	3.65	96.14

Where: $\bar{N_{A}}$—number of grains per unit area; $\bar{A}$—average area of the grain sections; * Grain Size Reduction calculated relatively to casting no. 1 grain size.

**Table 5:** Mechanical properties obtained for casting no. 1 (without RMF application—coarse grain) and casting no. 3 (RMF Frequency 75 Hz—fine grain).

Casting No.	Frequency (Hz)	Specimen No.	Yield Strength—Rp_0.2_ (MPa)	Engineering Ultimate Tensile Strength—Rm (MPa)	Elongation—A_5,01_ (%)
**1**	**0**	1	566.65	826.29	26.69
		2	570.78	824.84	32.57
		3	586.24	833.77	30.23
		**Average**	**574.56**	**828.30**	**29.83**
		**Std. Deviation**	**10.327**	**4.789**	**2.959**
**3**	**75**	1	572.88	836.33	26.19
		2	571.93	823.86	25.90
		3	579.72	837.98	30.31
		**Average**	**574.84**	**832.72**	**27.47**
		**Std. Deviation**	**4.252**	**7.717**	**2.468**

**Table 6:** Macro hardness measurements results (values presented in HV).

		Macro Hardness (HV)				
Casting No.	Frequency (Hz)	Meas. 1	Meas. 2	Meas. 3	Average	Standard Deviation
**1**	**0**	271	298	249	**272.67**	**24.54**
**2**	**15**	282	267	270	**273.00**	**7.94**
**3**	**75**	300	261	276	**279.00**	**19.67**
**4**	**150**	253	271	315	**279.67**	**31.90**

## Data Availability

Not applicable.
